# PARP Inhibitors: Clinical Limitations and Recent Attempts to Overcome Them

**DOI:** 10.3390/ijms23158412

**Published:** 2022-07-29

**Authors:** Dongha Kim, Hye Jin Nam

**Affiliations:** 1 Department of Anatomy, College of Medicine, The Catholic University of Korea, Seoul 06591, Korea; hidongha@catholic.ac.kr; 2 Drug Discovery Platform Research Center, Therapeutics and Biotechnology Division, Korea Research Institute of Chemical Technology, Daejeon 34114, Korea; 3 Department of Medicinal Chemistry and Pharmacology, University of Science and Technology, Daejeon 34113, Korea

**Keywords:** PARP1, resistance to PARP inhibitor, homologous recombination (HR), synthetic lethality, PROTAC, hydrophobic tagging

## Abstract

PARP inhibitors are the first clinically approved drugs that were developed based on synthetic lethality. PARP inhibitors have shown promising outcomes since their clinical applications and have recently been approved as maintenance treatment for cancer patients with BRCA mutations. PARP inhibitors also exhibit positive results even in patients without homologous recombination (HR) deficiency. Therapeutic effects were successfully achieved; however, the development of resistance was unavoidable. Approximately 40–70% of patients are likely to develop resistance. Here, we describe the mechanisms of action of PARP inhibitors, the causes of resistance, and the various efforts to overcome resistance. Particularly, we determined the survival probability of cancer patients according to the expression patterns of genes associated with HR restoration, which are critical for the development of PARP inhibitor resistance. Furthermore, we discuss the innovative attempts to degrade PARP proteins by chemically modifying PARP inhibitors. These efforts would enhance the efficacy of PARP inhibitors or expand the scope of their usage.

## 1. PARP Inhibitors for Cancer Treatment

Poly (ADP-ribose) polymerases (PARPs) are members of related enzymes that catalyze the transfer of ADP-ribose to target proteins [1,2]. PARP1 and PARP2 are considered to play an important role in maintaining genomic stability by mediating DNA repair processes. Among the PARP families, PARP1 shows abundant expression compared to the others and is responsible for most of the cellular PAR formation [3]; therefore, most studies have focused on PARP1. In this review, we discuss the mechanism of action of PARP inhibitors and the introduction of a new direction of therapeutic development utilizing PARP inhibitors, one of the important targeted anticancer therapies.

### 1.1. Synthetic Lethality of PARP Inhibitors

DNA damage response (DDR) is pivotal for maintaining genome stability because DNA is continually damaged by exogenous or endogenous genotoxic stress. It is estimated that DNA damage occurs at approximately 10,000 events per cell per day [4]. If damaged DNA is not swiftly recognized and properly corrected, genomic integrity is disrupted and various diseases, such as cancer, can occur. The repair machinery is specifically recruited according to the lesion of DNA damage; however, there is sufficient interactivity and redundancy to displace if certain pathways are disturbed or inhibited.

**Figure 1:**
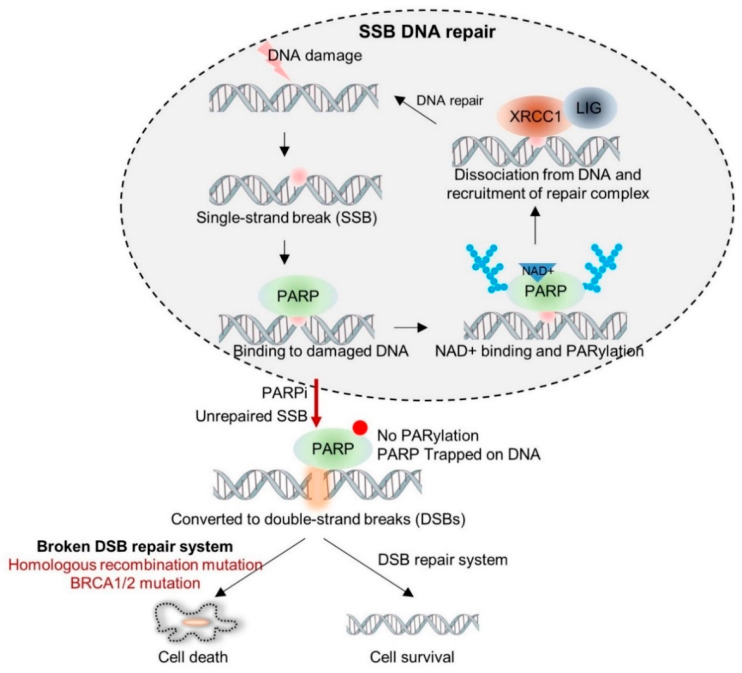
Synthetic lethality: mechanism of action of PARP inhibitors. PARPs recognize damaged DNA sites and recruit DNA repairing machineries through PARylation. Failure of repairing single-stranded DNA breaks can lead to DSB, which can be precisely repaired by the homologous recombination (HR) mechanism when the DNA repair system remains intact. However, cells with BRCA1/2 mutations progress to apoptosis. Because BRCA1/2 proteins play a key role in HR, the non-homologous end-joining (NHEJ) pathway is activated instead of HR in case of *BRCA1/2* mutated cells. Incorrect repair by NHEJ leads to genomic instability and eventually apoptosis.

PARPs recognize and bind to DNA damage and mediates poly(ADP-ribosyl)ation (also known as PARylation) of several target proteins, including itself. Auto-PARylation of PARPs recruits DNA-repair systems, such as XRCC1 and DNA ligase, and PARPs are dissociated from damaged DNA (Figure 1). PARPs are also involved in the base excision repair (BER) pathway [5]. The BER pathway involves removal of damaged bases by DNA glycosylase, cleavage of AP sites by AP endonuclease, replacement of the missing nucleotides by DNA polymerase β (POLβ), and ligation of the nicks by DNA ligase I (LIG1) or DNA ligase III (LIG3) [6]. Interestingly, PARPs are responsible for accelerating BER [7]. DNA double-strand breaks (DSB) are recognized by ATM or PARPs and are repaired through HR or non-homologous recombination (NHEJ) [8]. HR is an error-free repair process dependent upon the guidance of a homologous template, while NHEJ directly ligates the two broken DNA ends and often causes an alteration in the DNA sequences around the DSB sites. Persistent accumulation of incorrect repairs of DSBs destabilizes the genome and eventually leads to cell death.

Although PARPs are important for DDR and repair systems, the PARP1 knockout mouse model does not exhibit developmental defects or early onset of cancer. Continuous SSBs of DNA caused by PARP1 knockout lead to DSB, thus triggering the HR DNA repair system. In addition, induction of a compensatory DSB repair system or alternative role of PARP2 may explain the normal susceptibility of PARP1 knockout mice to cancer [9]. PARP1 is required for cellular recovery from DNA damage; therefore, DNA-damaging reagents can increase lethality when PARP1 is inhibited. Indeed, PARP1 knockout mice displayed hypersensitivity to gamma irradiation and alkylating reagents [9]. This concept supports combination therapy of PARP inhibitors.

BRCA1/2 proteins are involved in DNA DSB repair and plays an important role in accurate DNA repair through HR process. BRCA1/2 proteins are considered to be tumor suppressors. Numerous studies have revealed that mutations in *BRCA1/2* result in genomic instability and a high risk of developing ovarian and breast cancers; a breast cancer risk of 57% (95% confidence interval (CI), 0.47–0.66) for *BRCA1* and 49% (95% CI, 0.40–0.57) for *BRCA2* mutation carriers; and ovarian cancer risk of 40% (95% CI, 0.35–0.46) for *BRCA1* and 18% (95% CI, 0.13–0.23) for *BRCA2* mutation carriers [10]. It has been reported that approximately 5–10% of breast or ovarian cancer patients carry the *BRCA1/2* mutations [11]. Therefore, genetic testing has been increasing in individuals with a family history of breast or ovarian cancer. Prophylactic mastectomy and prophylactic oophorectomy are occasionally performed in individuals who have been identified with a *BRCA1/2* mutation through genetic testing. Multiple cases have reported that preventive surgery significantly reduces the risk of cancer [12,13].

Although inhibition of PARPs themselves does not significantly affect cell lethality, it can be fatal to cell viability if cells do not proceed to an alternative DNA repair system. Based on these findings, the idea of applying PARP inhibitors as a single agent to cancer patients with HR deficiency, such as *BRCA1/2* mutations, was derived. PARPs inhibition induces severe genomic instability in *BRCA1/2*-mutated cells, rendering the cells inviable [14,15], whereas PARPs inhibition does not affect normal cell viability. In other words, PARPs inhibition induces apoptosis when HR is defective. Two individual DNA repair pathways cooperate to maintain DNA integrity, and thus simultaneous inhibition of both pathways results in cell death, termed synthetic lethality, whereas inhibition of either pathway alone does not. 

### 1.2. PARP Inhibitors from Bench to Bedside

Various attempts have been made to demonstrate the concept of a synthetic lethal interaction between PARPs inhibition and *BRCA1/2* mutations in clinical trials. As PARPs use nicotinamide adenine dinucleotide (NAD^+^) as a substrate for processing PARylation, small-molecule nicotinamide analogs that compete with NAD^+^ to occupy the PARPs catalytic domain were initially used to prove the concept in preclinical assays [16].

**Figure 2:**
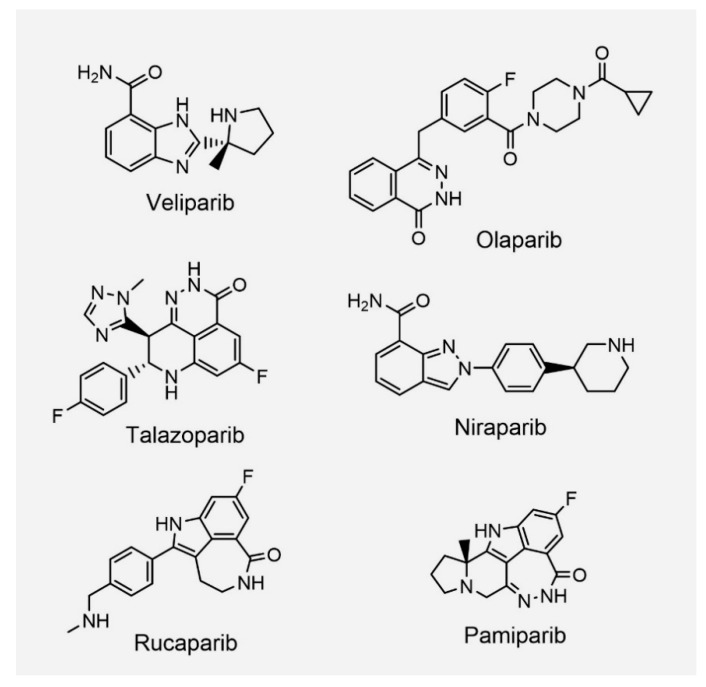
Chemical structures of representative PARP inhibitors.

Efforts to discover drugs have led to the development of clinically usable PARP inhibitors, including olaparib, veliparib, rucaparib, niraparib, talazoparib, and pamiparib (Figure 2 and Table 1) [16]. These PARP inhibitors interact with the NAD^+^ binding site. Enzymatic inactivation of PARPs and trapping of PARPs at damaged DNA are the mechanisms of action of PARP inhibitors [17].

In 2014, olaparib (Lynparza; AstraZeneca) was approved for use as a single reagent by the European Medicines Agency (EMA) in the European Union and by the USA Food and Drug Administration (FDA). The FDA and EMA approved olaparib for the treatment of patients with deleterious or suspected deleterious germline BRCA (gBRCA)-mutated advanced ovarian cancer who have been treated with three or more prior lines of chemotherapy [18]. Moreover, olaparib was approved for the maintenance treatment of adult patients with recurrent epithelial ovarian, fallopian tube, or primary peritoneal cancer, who had a complete or partial response to platinum-based chemotherapy in 2017 [19]. In 2018, olaparib was approved by the FDA for gBRCA mutated Her2-negative metastatic breast cancer. Among the PARP inhibitors, olaparib was the first to be approved for breast cancer treatment. In phase 3 clinical trials, the median progression-free survival (PFS) was significantly longer in the olaparib group than in the standard therapy group (7.0 months vs. 4.2 months; hazard ratio for disease progression or death, 0.58; 95% CI, 0.43–0.80) (OlympiAD trial, NCT02000622) [20]. As such, the clinical applications of olaparib have been increasing through various clinical trials and subsequent approvals. Notably, olaparib gained FDA approval in 2019 for the indication of pancreatic cancer (NCT02184195) [21]. Metastatic pancreatic cancer patients have been waiting for a long time for new therapeutic options. A phase 3 POLO trial (NCT02184195) showed that patients with a gBRCA mutation and metastatic pancreatic cancer that had not progressed during first-line platinum-based chemotherapy had significantly longer PFS with maintenance olaparib than with a placebo [21].

Rucaparib and niraparib (Zejula; GSK) have been approved for the treatment of ovarian cancer associated with HR deficiency. In addition, rucaparib has been approved for the treatment of patients with deleterious BRCA-mutated metastatic castrate-resistant prostate cancer (NCT02952534) [22]. Interestingly, the indications for niraparib were later expanded to patients with ovarian cancer regardless of their HR-deficient status. Significantly longer progression-free survival (PFS) in clinical trials (NCT02655016) led to FDA approval of niraparib as a maintenance therapy, regardless of biomarker status [23]. 

Veliparib (ABT-888; AbbVie) is undergoing phase 3 clinical trials as a combination therapy for breast, ovarian, and lung cancers. Although veliparib is an effective PARPs catalytic inhibitor with low IC_50_ values, its ability to trap PARPs is considered to be relatively weak [17]. However, veliparib increases sensitivity to treatments with DNA-damaging reagents, such as chemotherapy and radiation therapy [24]. A recent study reported that the median PFS in patients with gBRCA mutated and Her2-negative breast cancer increased when veliparib was added to carboplatine-paclitaxel treatment (veliparib: 16.6 months (95% CI 13.4–18.7) vs. control: 13.1 months (95% CI 11.4–14.5); NCT02163694) [25,26]. In addition, the combination with veliparib showed benefits in ovarian and lung cancers [27,28]. Interestingly, veliparib significantly improved the PFS of ovarian cancer patients regardless of the presence or absence of *BRCA* mutations or HR deficiency status, although the degree of benefit was higher in patients with HR deficiency [27]. Of note, this clinical trial included patients with BRCA1/2 somatic mutations. 

Talazoparib (BMN 673/Talzenna; Pfizer) was approved for advanced breast cancer patients with gBRCA mutations [29]. Talazoparib is known to exhibit the most potent PARPs trapping ability. It has a more rigid structure and is the largest in size among the PARP inhibitors [24,30]. It is thought that the difference in chemical structure results in the difference in the PARPs trapping ability. Of note, the PARPs trapping mechanism is controversial. The mere inhibition of catalytic activity, which inhibits PARylation-dependent dissociation, might stabilize PARPs on DNA [31]. Because the efficacy of the preclinical data may not always translate directly into clinical effectiveness, efficacy comparisons between PARP inhibitors in clinics are required in the future.

## 2. PARP Inhibitors: Back to the Bench: PARPi Resistance

### 2.1. Reversion Mutations of HR Genes 

Clinical trials have shown promising response rates in patients receiving PARP inhibitors; however, 40–70% of patients show a tendency to develop resistance over time. The molecular basis of acquired resistance has been increasingly elucidated. The most readily conceivable mechanism of PARP inhibitor resistance is the reversion mutation of *BRCA1/2*. In 2008, two independent research groups discovered BRCA reverse mutations that cause PARP inhibitor resistance [46,47]. Continuous exposure to PARP inhibitors or cisplatin ultimately resulted in resistance. Interestingly, it was confirmed that the protein-truncating c.6174delT frameshift mutation of *BRCA2* in cancer cells was converted to restore the open reading frame (ORF). Functional recovery of BRCA2 proteins caused resistance to PARP inhibitors. BRCA2 functionality was restored only by acquiring certain functional domains, even if it was not fully converted to the wild type (WT). Therefore, mapping the consequences of reversion mutations on BRCA proteins can reveal which protein domains are functionally important in conferring resistance to treatment. In patients, the primary *BRCA* genes mutations are mostly insertions or deletions subsequent to frameshifts and stop codon acquisitions. *BRCA1* primary mutations are predominantly located in the RING or BRCT domains [48], and *BRCA2* primary mutations are located in BRC repeats or the N-terminus [48]. Secondary reversion mutations occur where the primary mutation causes a frameshift and restores the ORF through new mutations. It is known that the BRCA1 RING domain functions as an E3 ligase when bound to BARD1, and the BRCT domain is critical for binding with phosphorylated proteins. Additionally, the BRCT domain is critical for the stabilization and nuclear localization of the BRCA1 protein [49]. BRCA2 binds with RAD51 recombinase, which is important for HR repair, through BRC repeats. Intriguingly, there have been no reports on reversion mutations in which all BRC repeats have been completely eliminated, suggesting that minimal BRC is required to restore resistance in conferring BRCA2 function [48]. However, some cancers without *BRCA* mutations are still sensitive to PARP inhibitors [50,51,52], suggesting that various other components are intricately involved in the HR process. 

In addition to BRCA reversion, secondary mutations of *RAD51C* and *RAD51D* were identified in patients administered rucaparib [53]. Interestingly, these mutations restored the function of RAD51 and increased resistance to PARP inhibitors [53]. These secondary mutations also cause clinical resistance to platinum-based chemotherapy.

### 2.2. Restoration of HR via Inactivation of Non-Homologous End Joining Proteins

Although both *BRCA1/2* reversion mutations have been identified in patients, BRCA2 reversions are more frequently reported; approximately four times as many reversion mutations in BRCA2 have been reported in clinics [48]. This suggests that in the case of the *BRCA1* mutation, there may be other mechanisms for inducing resistance to PARP inhibitors. BRCA1 is a pleiotropic DNA damage-responsive protein that is involved in both DNA repair and checkpoint activation, whereas BRCA2 is a mediator of the core mechanism of HR [54]. BRCA1 is responsible for multiple DDR steps—from the DNA damage sensor to the mediator, and finally to the repair effector. BRCA1 is phosphorylated by various checkpoint kinases, such as ATM, ATR, and CHK2, which regulate cell cycle checkpoints or facilitate DNA damage repair. In particular, BRCA1 functions upstream of BRCA2 in the BRCA1–BRCA2-mediated HR pathway [54]. Together, HR recovery and subsequent resistance induction in *BRCA1*-mutated cancers might be an outcome that bypasses multiple BRCA1-associated pathways.

BRCA1 is responsible for removing NHEJ proteins, such as p53-binding protein 1 (53BP1), from DSBs to prevent aberrant end-joining and to drive HR. Interestingly, in the absence of BRCA1, 53BP1 suppresses HR by limiting DNA end resection, which is critical for excessive NHEJ and subsequent apoptosis [55,56]. Therefore, it is exceedingly likely that 53BP1 inactivation affects the choice between NHEJ and HR, and, ultimately, the resistance. Indeed, loss of 53BP1 partially restores HR in BRCA1-deficient cells and develops resistance to PARP inhibitors [57]. Since 53BP1 expression is frequently lost in *BRCA1/2*-mutated or triple-negative breast cancers [58], it is thought that 53BP1 could be used as a biomarker for predicting the response of *BRCA*-mutated cancers to PARP inhibitor therapy. Consistently, 53BP1-negative breast cancer patients showed a poorer survival rate [58]. Loss of 53BP1 also develops resistance to DNA-damaging reagents, such as cisplatin and doxorubicin [57]. 

**Figure 3:**
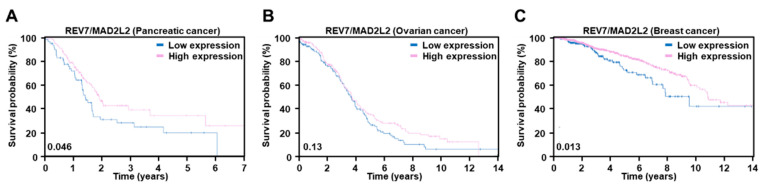
Kaplan–Meier plots for *REV7/MAD2L2* mRNA level separation from TCGA and HPA cohorts in pancreatic cancer (**A**), ovarian cancer (**B**), and breast cancer (**C**). The log-rank *p* values are shown in the lower left corner of each Kaplan–Meier plot. The prognosis of each group of patients was examined by Kaplan-Meier survival estimators, and the survival outcomes of the two groups were compared by log-rank tests. To choose the best fragments per kilobase of exon per million (FPKM) cut-offs for grouping the most significant patients, all FPKM values from the 20th to 80th percentiles were used to group the patients, significant differences in the survival outcomes of the groups were examined, and the value yielding the lowest log-rank *p* value selected. Genes with log-rank *p* values less than 0.001 were defined as prognostic genes.

Loss of REV7/MAD2L2 also leads to HR restoration and subsequent PARP inhibitor resistance in BRCA1-deficient cells [59]. REV7/MAD2L2 is recruited to DNA damaged sites as a downstream of 53BP1 and blocks DNA resection to promote NHEJ [59]. Therefore, REV7/MAD2L2 depletion drives BRCA1-deficient cells to repair via HR pathways and renders them resistant to PARP inhibitors. The correlation of survival rates according to the degree of expression of *REV7/MAD2L2* was investigated. The TCGA database was used to construct gene-specific survival probability panels for three types of cancers (pancreatic cancer, ovarian cancer, and breast cancer) where PARP inhibitors are predominantly used for treatment. High expression of *REV7/MAD2L2* shows higher survival probabilities in pancreatic cancer (Figure 3A), ovarian cancer (Figure 3B), and breast cancer (Figure 3C), whereas low expression of *REV7/MAD2L2* exhibits an unfavorable outcome (Figure 3).

### 2.3. Restoration of Replication Fork Stability 

Timely and faithful duplication of the genome should be performed for genomic stability. Occasionally, impediments to the replication progress, such as DNA lesions or collisions with transcription machineries, lead to fork slowdown and/or stalling known as replication stress. PARP and BRCA play key roles in DNA replication, particularly for fork protection [60]. Multiple fork protection mechanisms, consisting of stabilization, repair, and restart processes, minimize genomic instability [60]. Paradoxically, these mechanisms are also active in cancer cells; however, they only serve to compromise the cytotoxicity caused by therapeutic agents. PARP inhibitors appear to increase replication fork speed and induce accumulated single-strand DNA damage and subsequent DSB [61]. These replication barriers caused by PARP inhibitors lead to fork stalling. BRCA proteins prevent the degradation of nascent DNA after replication stress; therefore, nascent DNA generated from stalled forks is degraded by MRE11 nuclease in BRCA-deficient cells, resulting in excessive genomic instability and apoptosis; that is, restoring fork stabilization in BRCA-deficient cells may be responsible for triggering PAPR inhibitor resistance, and a comprehensive understanding of how cells protect stalled forks could lead to the establishment of strategies to combat PARP inhibitor resistance in cancer treatment [55,60,62,63,64].

**Figure 4:**
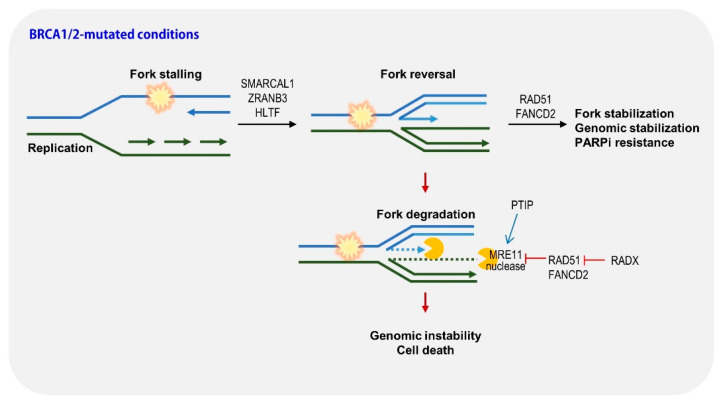
Restoring fork stabilization can render resistance to PARP inhibitors.

**Figure 5:**
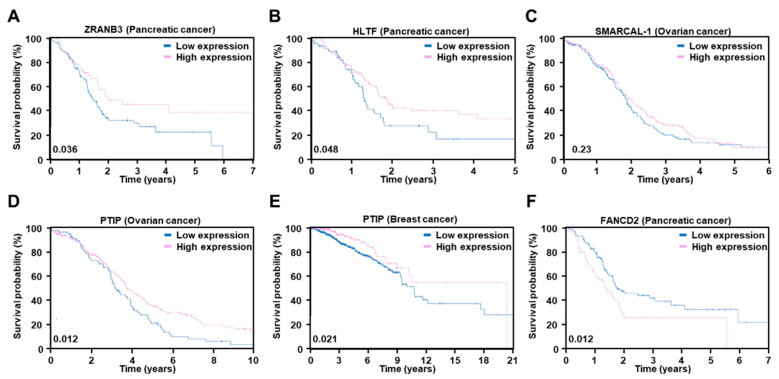
Kaplan–Meier plots for the *ZRANB3* in pancreatic cancer (**A**), *HLTF* in pancreatic cancer (**B**), *SMARCAL-1* in ovarian cancer (**C**), *PTIP* in ovarian cancer (**D**) or breast cancer (**E**), and *FANCD2* in pancreatic cancer (**F**) mRNA level separation from the TCGA and HPA cohorts. The log-rank *p* values are shown in the lower left corner of each Kaplan–Meier plot. The prognosis of each group of patients was examined by Kaplan–Meier survival estimators, and the survival outcomes of the two groups were compared by log-rank tests. To choose the best fragments per kilobase of exon per million (FPKM) cut-offs for grouping the most significant patients, all FPKM values from the 20th to 80th percentiles were used to group the patients, significant differences in the survival outcomes of the groups were examined, and the value yielding the lowest log-rank *p* value selected. Genes with a log-rank *p* values less than 0.001 were defined as prognostic genes.

Interestingly, numerous studies have reported that restoring fork stabilization can render resistance to PARP inhibitors (Figure 4). Through impairment of MRE11 nuclease recruitment by MLL3/4 depletion, PTIP restores fork stability and drives resistance to PARP inhibitors [63]. Inactivation of SNF2-family fork remodelers, including SMARCAL1, ZRANB3, and HLTF, which are critical for fork reversal, reduces replication stress and genomic instability, and provides PARP inhibitor resistance [64]. RADX (CXorf57), a single-stranded DNA-binding protein, regulates RAD51 activity. RADX depletion restores fork protection in *BRCA* mutant cells and induces PARP inhibitor resistance [65]. Consistently, studies using *BRCA*-mutated cells with low expression of *PTIP, RADX*, and *SMARCAL-1* showed poorer survival outcomes in a xenograft mouse model [60,63,64,65]. We also constructed survival probability panels for these genes using the TCGA database. In pancreatic cancer, similar to *REV7/MAD2L2*, low expression of *ZRANB3* and *HLTF* shows poorer survival probabilities than those with high expression (Figure 5A,B). In ovarian cancer, low expression of *SMARCAL-1* and PTIP exhibits an unfavorable outcome (Figure 5C,D). *PTIP* also shows similar survival probabilities in breast cancer (Figure 5E). FANCD2 overexpression confers resistance to PARP inhibitors through fork stabilization, suggesting that FANCD2 can be used as a biomarker for PARP inhibitors sensitivity [62]. Indeed, high expression of *FANCD2* shows a lower survival probability in pancreatic cancer (Figure 5F). Surprisingly, stalled fork stabilization conferred resistance to PARP inhibitors and to chemotherapy such as cisplatin. As mentioned above, HR restoration also conferred resistance to chemotherapy. Taken together, these results suggest that resistance can be acquired through alteration of the DNA repair system in the case of DNA damage-related therapeutics. 

### 2.4. PARP Mutations 

PARP inhibitors target PARP proteins; therefore, the alteration of the PARP protein itself might lead to resistance. Mutation or depletion of target proteins can affect resistance to PARP inhibitors. *PARP1* knockout cells were highly resistant to olaparib treatment [17], suggesting that PARP1 loss might lead to resistance. Genome-wide CRISPR–Cas9 mutagenesis screening identified several mutations in PARP1 that are related to PARP inhibitor resistance [66]. *PARP1* mutations located in the DNA-binding ZnF domain failed to be recruited to the DNA damage site and did not produce PAR at damaged sites. Interestingly, ovarian cancer patients with the *PARP1* p.R591C mutation (c.1771C>T) showed de novo resistance to olaparib, suggesting that this is partly validated in clinics as a mechanism of resistance to PARP inhibitors. Although the p.R591C mutation is not located in the ZnF domain, the dissociation rate from the damage sites is more rapid than that of WT. 

## 3. Lessons from Bench: How Can We Overcome Resistance to PARPi or Expand the Use of PARPi?

### 3.1. PARPi Combination Approaches

Combination with inhibitors of particular cell cycle checkpoint kinases can enhance PAPR inhibitor sensitivity. Replication stress activates multiple cell cycle checkpoint kinases. The kinase ATR primarily recognizes a stalled replication fork and phosphorylates downstream signaling cascades, including BRCA1, Mec1, and 53BP1. Activated ATR induces a shutdown of origin firing and lowers fork speed through the activation of CHK1 and inactivation of CDK1/2 [67,68,69]. WEE1 kinases phosphorylate CDK1/2 to maintain their inactivation [70]. In other words, activation of ATM and WEE1 contributes to the maintenance of genomic stability in normal cells. ATR kinase plays a unique role in PARP inhibitor-resistant cells [55]. In PARP inhibitor-resistant BRCA1-deficient cells, ATR regulates both BRCA1-independent HR and fork protection by promoting RAD51 recruitment to DSBs and stalled forks [55]. These results indicate that PARP inhibitor resistance in BRCA1-deficient cancers might be overcome by co-treatment with an ATR inhibitor. Based on these results, phase 2 clinical trials of combination therapy with an ATR inhibitor (AZD6738) and olaparib for solid cancers, such as renal, pancreatic, and breast cancers (NCT03682289, NCT03330847), are ongoing. In a similar context, WEE1 inhibitors have emerged as promising alternatives, and various clinical trials are underway to evaluate their efficacy. Among them, phase 1 clinical trials are recruiting advanced solid cancer patients with selective mutations (*BRCA1*, *BRCA2*, *BRIP1*, *FANCA*, *PALB2*, or non-DDR gene markers) and PARP resistance (NCT04197713, STAR study). We anticipate that the results of overcoming PARP inhibitor resistance by WEE1 inhibitor (adavosertib/AZD1775) will be reproduced in a clinical setting.

Blocking CTLA4, PD-1, and PD-L1 immune checkpoints have emerged as new targets in cancer therapy, and these therapies have shown remarkable positive clinical effects. Interestingly, PARP inhibitor treatment upregulated PD-L1 expression in breast cancer cells, which weakened the efficacy of PARP inhibitors in terms of anti-cancer immunity [71]. An in vivo study showed that the combination of PARP inhibitors and anti-PD-L1 therapy significantly increased the therapeutic efficacy [71]. Currently, phase 2 clinical trials using olaparib and anti-PD-L1 (durvalumab or atezolizumab) combination for ovarian, breast, and gastric cancers (NCT02734004, NCT02849496) are ongoing.

Combination with inhibitors against restoration of PARylation can enhance PAPR inhibitor sensitivity. It has been reported that PARP1 phosphorylation at Tyr907 by c-Met increases PARylation activity of PARP1 and renders cancer cells resistant to PARP inhibitor [72]; that is, pY907 PARP1 might be a predictive marker for PARP inhibitor resistance. Indeed, the combination of c-Met and PARP1 inhibitors reduced tumor growth compared to either inhibitor alone. Loss of PAR glycohydrolase (PARG) is also related to restoration of PARylation. PARG degrades PAR by hydrolyzing the ribose–ribose bonds in poly(ADP-ribose) [73]. PARG knockdown partially restored PARylation despite PARP inhibitor treatment and resulted in PARP inhibitor resistance [50]. Interestingly, alteration of PAR signaling by downregulation of PARG increases the sensitivity to IR [50,52]. These studies suggest that assessment of PARG or c-Met activation is required to establish an appropriate therapeutic strategy. 

Together, combination therapies to overcome PARP inhibitor resistance and enhance PARP inhibitor sensitivity are still in their infancy and have a long way to go. An increasing number of studies are required to investigate its feasibility in clinical settings.

### 3.2. Chemical Modifications of PARP Inhibitors

Researchers have attempted to develop more potent and broadly useful PARP inhibitors. Recently, chemically mediated targeted protein degradation has emerged as a promising and innovative technology for the development of new therapeutic drugs. Removal of targeted protein degradation through the ubiquitin proteasome system or autophagy generally provides a more potent inhibitory ability. The most well-known modulation methods are proteolysis-targeting chimeras (PROTAC) and hydrophobic tagging. PROTAC has the advantage of being easy to target intracellularly. Furthermore, it can be used to develop a therapeutic agent for intractable diseases because it can remove disease proteins that conventional antibody therapeutics have not been able to access.

Recently, the clinical application of the PROTAC method for cancer treatment has been attempted in various ways. In the second half of 2020, three PROTAC-related companies were listed in the US, with Arvinas announcing the positive results of their phase 1 clinical pipeline in December 2020, raising interest in PROTAC-related anticancer drugs [74]. In 2019, clinical trials were sequentially initiated for ARV-110, a metastatic castration-resistant prostate cancer treatment, and ARV-471, a locally advanced or metastatic ER-positive/HER2-negative breast cancer treatment. In addition, in October 2019, the initial safety and pharmacokinetic data of ARV-110 and ARV-471 were published (NCT03888612, NCT04072952). Recently, Arvinas announced the interim results of a phase 1 clinical trial and reported that the safety and tolerability problems of PROTAC were solved to a certain extent. However, it is still in the early stages of clinical research; therefore, it is necessary to broaden the scope of applied research, such as targeting PARP and PARP inhibitors. 

**Figure 6:**
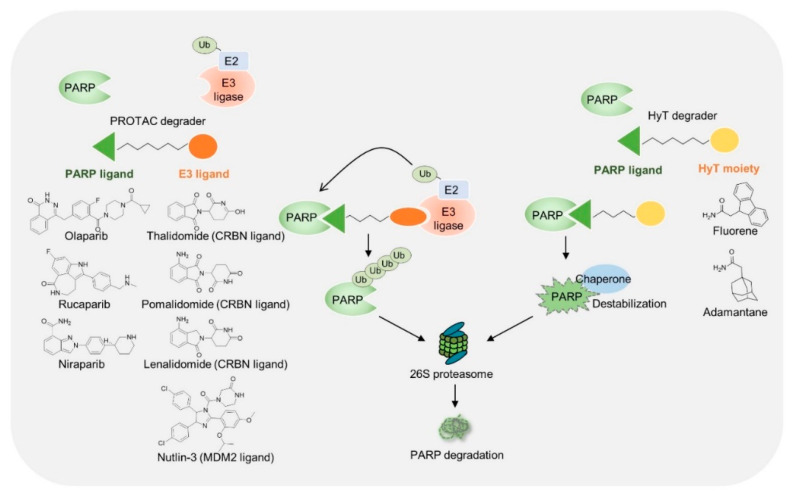
PARP degradation by proteolysis-targeting chimeras (PROTAC) or hydrophobic-tagged small molecules. Chemical structures used in PARP degradation are shown. Ub, ubiquitin; E3, E3 ligase.

To develop PARP-targeting PROTAC, a PARP inhibitor was connected to the E3 ubiquitin ligase ligand through a flexible linker (Figure 6). This bivalent molecule brings the E3 ligase and PARPs into close proximity, where PARPs are ubiquitinated and subsequently degraded through the proteasome pathway. Although more than 600 E3 ligases are expressed in mammalian cells, only limited E3 ligases (e.g., MDM2, cIAP1, cereblon [CRBN], VHL, RNF4, RNF14, and DCAF16) are used for PROTAC [75]. The choice of pairing E3 and PARP binders appears to be critical for the successful degradation of target proteins by PROTAC. In addition, the linker length should be optimized to allow ternary complex formation between E3 ligase and target proteins [76]. PROTACs using olaparib, rucaparib, and niraparib derivatives were developed and tested in cancer and non-cancer cells (Table 2). They showed superior cytotoxicity compared to that of conventional inhibitors and a wider range of uses beyond *BRCA* mutant cancers [77,78,79]. Among them, PROTAC using niraparib and MDM2 ligand exhibited PARP1 degradation and also increased PARP cleavage in MDA-MB-231 breast cancer cells. An interesting study was conducted to uncouple PARP1 catalytic inhibition and PARP1 trapping through the development of PARP PROTAC [80,81]. As mentioned above, PARP inhibitors can inhibit catalytic activity and induce PARPs trapping. PROTAC using rucaparib and CRBN ligand (iRucaparib-AP6) selectively degraded PARP1 and inhibited PARylation-mediated signaling events [81]. Interestingly, iRucaparib-AP6 did not induce PARP1 trapping or cell death in cardiomyocytes. Non-trapping PARP degraders are expected to be useful for the treatment of diseases associated with PARP activation (e.g., ischemia–reperfusion injury or neurodegenerative disease) because it does not induce genotoxicity or cell death.

Hydrophobic-tagging technology consists of a hydrophobic fragment (e.g., adamantyl, Boc3Arg, or fluorene) and a ligand for the targeted protein. Hydrophobic tagging mimics a partially unfolded or misfolded protein state and induces protein destabilization, thereby recruiting an endogenous chaperone protein to a hydrophobic-tagged protein and then degrading the targeted protein by the proteasome (Figure 6). Hydrophobic-tagged olaparib showed enhanced apoptosis in MBA-MB-231 cells, which are triple-negative breast cancer cells and carry the BRCA WT [82]. Interestingly, hydrophobic-tagged olaparib triggered the unfolded protein response (UPR) and endoplasmic reticulum (ER) stress. Excessive and irreversible UPR and ER stress-mediated apoptosis by hydrophobic tagging might be a mechanism of action that has better antitumor effects than olaparib in TNBC cells.

**Figure 7:**
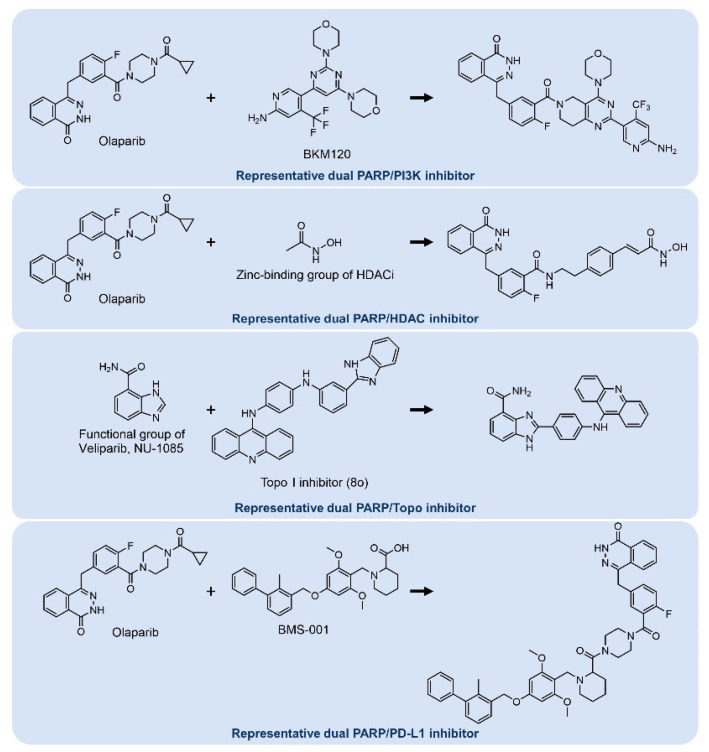
Chemical structures of representative dual inhibitors. Dual inhibitor development for improved outcomes. Concomitant inhibition of PARP and other pathways is achieved by linking two bioactive molecules.

Hybrid drugs (also known as single-molecule multiple targets) also provide synergistic effects in treatment. Hybrid drugs are obtained by connecting two or more bioactive molecules [83,84]. Hybrid drugs have been developed based on combination therapies that overcome resistance or show improved outcomes, and their efficacy has been evaluated in preclinical studies (Figure 7).

As several BRCA1-related breast cancers are associated with activation of PI3K signaling [85], dual inhibitors targeting PARP and PI3K can be a promising strategy for cancer treatment. Based on this concept, dual inhibitors targeting PARP and PI3K were developed, and a more enhanced dual targeting capability was observed in cell-based assays [86]. Interestingly, dual inhibitors were found to be more efficacious than single administration of olaparib or BKM120 (PI3K inhibitor) and combined administration of olaparib and PI3K inhibitor in xenograft mouse models [86]. As co-treatment with HDAC inhibitors and PARP inhibitors exhibited synergistic effects in various cancer cells [87,88], researchers developed drugs that can simultaneously target PARP and HDAC. Dual PARP and HDAC inhibitors showed excellent inhibitory activities against PARP and HDAC and induced apoptosis in breast cancer cells [89]. DNA topoisomerases are involved in resolving the topological issues caused during replication or transcription [90]. Since both PARP inhibitors and topoisomerase I/II inhibitors are involved in replication fork stabilization, combinatorial inhibition of both target proteins may help overcoming resistance. Dual PARP and topoisomerase inhibitors showed significant anti-tumor activity without adverse toxicity in xenograft mouse models [91]. Hybrid inhibitors of PARP and PD-L1 were also developed by researchers. It has been reported that anti-tumor activity is improved when a PARP inhibitor is combined with a PD-1 antibody (pembrolizumab). In addition, PARP inhibitor treatment has been reported to upregulate PD-L1 expression in some cancer cells [92]. The dual inhibitors not only showed anti-tumor activity but also attenuated PD-L1 expression compared to the PARP inhibitor alone [93].

## 4. Concluding Remarks

Extensive efforts have been made to develop efficient PARP inhibitors, and drugs with high efficacy and tolerable toxicity have been actively used in clinics. Indeed, PARPs are undoubtedly an excellent drug target applicable to patients with *BRCA* mutations, and its scope of application is currently being extended. However, the occurrence of resistance is inevitable owing to the inherent limitations of targeted therapy. As seen above, except for the reversion mutation of *BRCA*, it appears that certain clues have been identified that show resistance can be overcome through preclinical assays, although clinical evidence is still lacking. 

Although combination approaches that identify the mechanism of resistance and provide solutions are the forerunners, it is thought that the development of more creative and newer concepts of PARP inhibitors is required to prepare for unexpected situations in the future. The application of new technologies such as PROTAC or hydrophobic tagging to existing inhibitors is an impressive endeavor, although it is still in its infancy. As degrader technology continues to mature and be optimized, promising results are expected to be achieved in the near future. The development of structurally diverse inhibitors may be another option. To date, the majority of PARP inhibitors are NAD^+^ competitors, and their mechanism of action is almost similar. A recent study suggested structurally new non-NAD-like inhibitors and showed greater efficacy and potency than classical PARP inhibitors [94]. If innovative explorations are continuously performed and accumulated, potent drugs with new mechanisms are expected to be developed and used for patients.

## Figures and Tables

**Table 1:** Notable phase 3 and 4 clinical trials that have results.

NCT Identifier	Drug	Setting Conditions	With Chemo	Efficacy (Ref.)
Breast cancer				
NCT02163694	Veliparib	Her2-negative advanced breast cancer; Germline *BRCA1/2* mutated	Yes; Combination with carboplatin + paclitaxel	Increase in PFS compared with placebo in a germline BRCA1/2 mutation [25,26]
NCT02000622	Olaparib	Her2-negative metastatic breast cancer patients; Germline *BRCA 1/2* mutated	No	Benefit over standard chemotherapy in PFS [20,32]
NCT01945775	Talazoparib	Advanced or metastatic breast cancer; Germline *BRCA* mutated	No	Benefit over standard chemotherapy in PFS [29,33] Approved by U.S. FDA in 2019
NCT01905592	Niraparib	Advanced or metastatic breast cancer	No	No significant differences between niparparib and standard chemotherapy in PFS and OS
Ovarian cancer				
NCT01847274	Niraparib	Ovarian cancer; platinum-based chemotherapy sensitive	No	Increase in PFS compared with placebo regardless of the presence or absence of HRD [23,34,35]
NCT02655016	Niraparib	Advanced ovarian cancer (Stage III or IV); Patients with clinical complete response or partial response following completion of platinum-based chemotherapy course.	No	Increase in PFS compared with placebo regardless of the presence or absence of HDR [36] Approved by U.S. FDA in 2020 for the maintenance treatment
NCT02470585	Veliparib	Advanced ovarian cancer (Stage III or IV); Patients after surgery	Yes; Combination with first-line chemotherapy	Increase in PFS compared with placebo regardless of the presence or absence of HRD [27]
NCT01968213	Rucaparib	Ovarian, fallopian, peritoneal cancer; Patients with clinical complete response or partial response following completion of platinum-based chemotherapy course.	No	Increase in PFS, CFI, TFST, TSST, and PSF2 compared with placebo in recurrent ovarian cancer regardless of the presence or absence of HDR [37,38,39] Approved by U.S. FDA in 2018 for the maintenance treatment
NCT01874353	Olaparib	Relapsed high grade serous ovarian cancer; platinum-based chemotherapy sensitive; *BRCA1/2* mutated	No	Increase in PFS and OS compared with placebo [40,41,42,43] Approved by U.S. FDA in 2017 for the maintenance treatment
NCT01844986	Olaparib	Advanced Ovarian Cancer (Stage III, IV); *BRCA1/2* mutated; Patients with clinical complete response or partial response following completion of platinum-based chemotherapy course.	No	Increase in PFS compared with placebo regardless of the presence or absence of HRD [44,45]
Lung cancer				
NCT02106546	Veliparib	Advanced or metastatic squamous non-small cell lung cancer (NSCLC);	Yes; Combination with carboplatin + paclitaxel	Favorable OS in the 52-gene expression histology classifier (LP52)-positive population by veliparib; Favorable OS in the LP52-negative population by placebo [28]
Pancreatic cancer				
NCT02184195	Olaparib	Metastatic pancreatic cancer; Germline *BRCA1/2* mutated; No progression during first-line platinum-based chemotherapy	No	Increase in PFS compared to placebo with a germline BRCA1/2 mutation [21] Approved by U.S. FDA in 2019 for the maintenance treatment

**Table 2:** PROTAC or hydrophobic-tagged PARP inhibitors.

PROTAC				
PARP Binder	E3 Ligase Binder	Tested Cell	Note	Ref.
Olaparib	CRBN ligand	MDA-MB-436 (*BRCA1* mutated breast cancer cells), Capan-1 (*BRCA2* mutated pancreatic cancer cells), SW620 (colon cancer cell)	Inhibition of tumor growth, Xenograft assay	[77]
Rucaparib	CRBN ligand	Primary rat neonatal cardiomyocytes, C2C12 (myoblast)	PARP1 non-trapping, No genotoxic induced cell death	[81]
Olaparib	CRBN ligand	SW620	Increased apoptosis	[78]
Niraparib	MDM2 ligand	MDA-MB-231 (TNBC)	Induction of PARP1 cleavage, increased apoptosis	[79]
**Hydrophobic Tagging**				
**PARP Binder**	**Hydrophobic Moiety**	**Tested Cell**	**Note**	**Ref.**
Olaparib	Fluorene	MDA-MB-231, MDA-MB-468 (TNBC), HCC1937 (*BRCA1* mutated breast cancer cells)	Increased apoptosis and ER stress	[82]

## Data Availability

Not applicable.
